# A hypothesis to explain accuracy of wasp resemblances

**DOI:** 10.1002/ece3.2586

**Published:** 2016-12-05

**Authors:** Michael Boppré, Richard I. Vane‐Wright, Wolfgang Wickler

**Affiliations:** ^1^ Forstzoologie und EntomologieAlbert‐Ludwigs‐UniversitätFreiburgGermany; ^2^ Durrell Institute of Conservation and Ecology (DICE)University of KentCanterburyUK; ^3^ Life SciencesNatural History MuseumLondonUK; ^4^ Max‐Planck‐Institut für OrnithologieSeewiesenGermany

**Keywords:** adaptive resemblance, crypsis, evolution, learned/innate responses, masquerade, mimesis, Müllerian/Batesian/imperfect/inaccurate mimicry, selecting agents, wasps, Diptera, Syrphidae, hoverflies, Hymenoptera, Vespidae, Lepidoptera, Arctiinae, moths

## Abstract

Mimicry is one of the oldest concepts in biology, but it still presents many puzzles and continues to be widely debated. Simulation of wasps with a yellow‐black abdominal pattern by other insects (commonly called “wasp mimicry”) is traditionally considered a case of resemblance of unprofitable by profitable prey causing educated predators to avoid models and mimics to the advantage of both (Figure 1a). However, as wasps themselves are predators of insects, wasp mimicry can also be seen as a case of resemblance to one's own potential antagonist. We here propose an additional hypothesis to Batesian and Müllerian mimicry (both typically involving selection by learning vertebrate predators; cf. Table 1) that reflects another possible scenario for the evolution of multifold and in particular very accurate resemblances to wasps: an innate, visual inhibition of aggression among look‐alike wasps, based on their social organization and high abundance. We argue that wasp species resembling each other need not only be Müllerian mutualists and that other insects resembling wasps need not only be Batesian mimics, but an innate ability of wasps to recognize each other during hunting is the driver in the evolution of a distinct kind of masquerade, in which model, mimic, and selecting agent belong to one or several species (Figure  1b). Wasp mimics resemble wasps not (only) to be mistaken by educated predators but rather, or in addition, to escape attack from their wasp models. Within a given ecosystem, there will be selection pressures leading to masquerade driven by wasps and/or to mimicry driven by other predators that have to learn to avoid them. Different pressures by guilds of these two types of selective agents could explain the widely differing fidelity with respect to the models in assemblages of yellow jackets and yellow jacket look‐alikes.

## 1 “Wasp Mimicry”

With their conspicuous yellow‐black striped abdomens, worker wasps of, for example, *Vespula* and *Dolichovespula* (yellow jackets *s.str*.; Vespinae) are highly aposematic. Worldwide, a considerable number of wasp species (yellow jackets *s.l*.) belonging to the Vespidae (Hymenoptera), including Polistinae (paper wasps: e.g., *Mischocyttarus, Agelaia*), share yellow jacket features. These eusocial wasps are among the best defended insects: They have powerful stings that can be used to inject venom into an invader, making them unprofitable prey for all but the most specialized predators. Many co‐occurring, well‐defended species of wasps closely resemble one another, and the respective species are considered to gain from mutualistic Müllerian mimicry (Archer, 2012; Müller, 1878; Richards, 1978).

**Figure 1:**
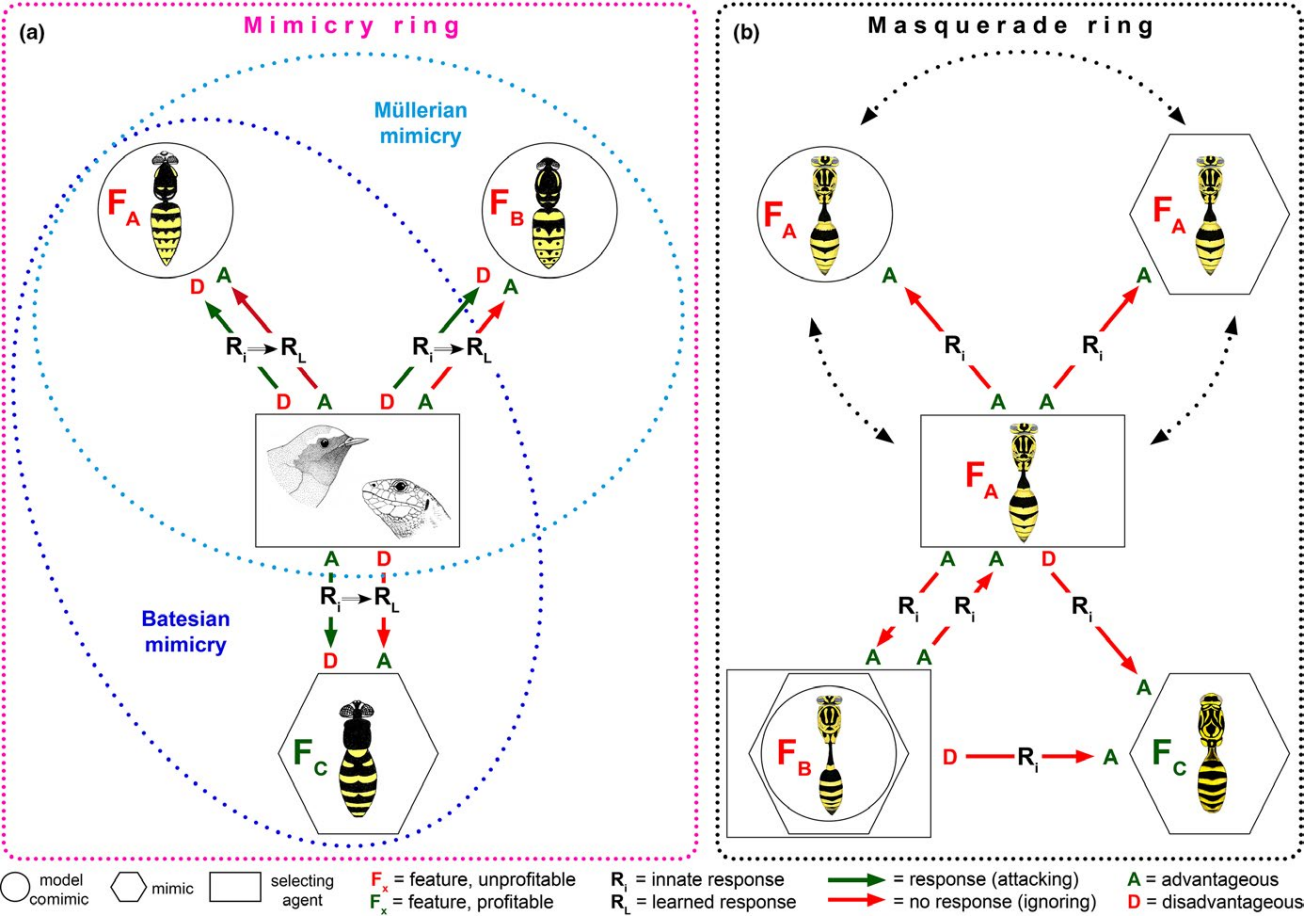
(a) Current interpretation of “wasp mimicry” as Batesian and Müllerian mimicry in response to a guild of vertebrate insectivores acting as selecting agents able to learn by experience. Evolution of the yellow jacket pattern (F) that warns the potential predators that wasps are unprofitable prey, which they learn to avoid, together with evolution of close resemblance between two or more wasp species as a result of the mutual selective advantages of pattern standardization (Müller's hypothesis; F_A_ = F_B_). Once such an effective warning pattern has been established, profitable prey can take advantage by evolution of a simulated yellow jacket pattern (F_C_) that is sufficiently similar to the wasp pattern that at least some of the insectivores reject them on sight (Bates’ hypothesis). In this scheme, depicting a European *Vespula* mimicry ring, the innate response (R_i_) of an inexperienced insectivore is to attack any potential insect prey. In the case of a vespid wasp, this innate response is a disadvantage (D) to predator and wasp alike. Through bad experience and associative learning, the predator learns not to attack yellow jackets. This learned response (R_L_) is then an advantage (A) to predators and wasps alike, making it possible for profitable prey (F_C_) that are sufficiently similar in outward appearance to elicit the learned response, and thereby escape from attack. (b) Our additional interpretation of yellow jacket wasp mimicry is based on innate recognition of nestmates plus non‐aggression towards foraging individuals of the same and other eusocial vespids, all visually mediated by their conspicuous and very similar appearance. Once such a system is functional, accurate yellow jacket appearance evolved by otherwise profitable prey insects can protect them from predation by wasps. Such insects benefit by simulation of their own potential predators. In the scheme, presented for a Costa Rican *Agelaia* masquerade ring, three sister wasps F_A_ (with yellow jacket pattern) symbolize roles equivalent to model, mimic, and selecting agent: in reality, each wasp, by means of its standard appearance and inhibitory response to its own specific pattern, performs all three roles simultaneously and interchangeably. Another social wasp species with a very similar pattern (F_B_), in which each individual (as in F_A_) is model, mimic, and selecting agent (as symbolized by the nested circle, polygon, and square), responds to its own nestmates and to F_A_ wasps in the same way, as do F_A_ wasps to F_B_ wasps. All wasps benefit (A) from this mutual inhibition of intra‐ and interspecific aggression towards equally dangerous and well‐defended community members. Establishment of such communication makes it possible for profitable prey very similar in outward appearance (F_C_) to elicit the innate wasp–wasp response, and thereby avoid being attacked. This is an advantage (A) to potential prey but a disadvantage (D) to the wasps. All responses in this system are innate (Ri), with no learning involved, thus falling outside the scope of Batesian as well as Müllerian mimicry (Figure 1a)

Undefended insects (profitable prey) of several orders simulate more or less accurately the conspicuous features (color pattern, size, shape, flight, sound) of yellow jackets, and this phenomenon is widely considered to fit the classic concept of Batesian mimicry (Bates, 1862). Thus, the totality of the ‘yellow jacket warning coloration assemblage’ seems to represent a true classical mimicry ring consisting of both Müllerian and Batesian mimics in concert (Figure 1a). A few antiselectionists argued strongly against wasp mimicry; notably, they compiled various observations and experiments showing that wasps (and their mimics) may be less avoided by vertebrates than is widely assumed (e.g., Haase, 1893; Heikertinger, 1921; Mostler, 1935). Regardless of details, because yellow‐black wasps occur commonly in almost all ecosystems, including conurbations, and frequently make people feel annoyed, “wasp mimicry” has become a prime example about which one can read not only in textbooks but also in many publications intended for the general public. Needless to say, yellow and black color patterns are alerting signals in general, common in nature (in fish, salamanders, frogs, caterpillars, even “high‐visibility jackets” worn by emergency service personnel) and, of course, not only associated with yellow jacket wasps.

## 2 Diverse Fidelity of Simulated Features Poses Questions

A recently much debated problem is the occurrence of “imperfect” (= “inaccurate”) mimicry (e.g., Edmunds, 2000; Gilbert, 2005; Johnstone, 2002; Pekár, Jarab, Fromhage, & Herberstein, 2011; Penney, Hassall, Skevington, Abbott, & Sherratt, 2012; Pfennig, 2012; Pfennig & Kikuchi, 2012; Sherratt, 2002). While in some cases of mimicry *s.l.,* there is hardly any variation in fidelity of the simulated entities or features (e.g., pheromones: Stowe, 1988; Dettner & Liepert, 1994), in others fidelity between models and mimics varies widely. Several hypotheses have been proposed to explain variation in fidelity of mimics (see Kikuchi & Pfennig, 2013; Pfennig & Kikuchi, 2012).

**Figure 2:**
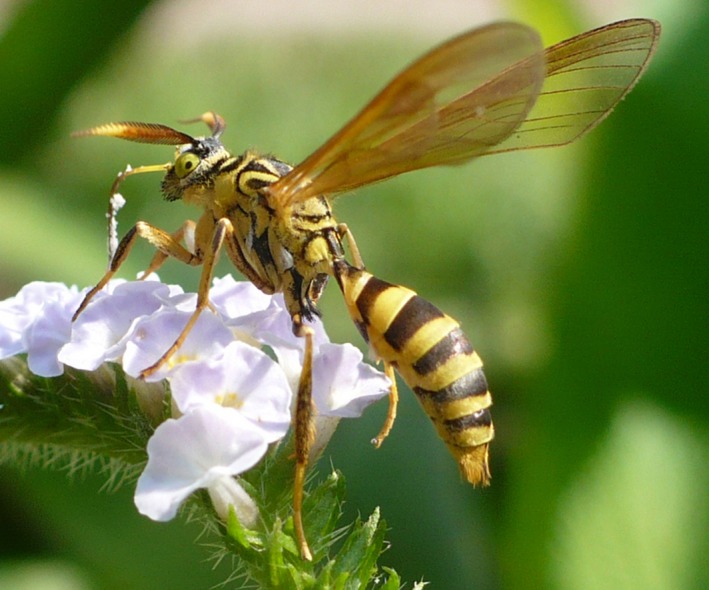
Not a stinging wasp but a harmless day‐flying moth (Lepidoptera: Erebidae: Arctiinae: *Pseudosphex laticincta*). These moths are “sheep in wolves’ clothing” and simulate their predators—this is not necessarily a case of classical mimicry. Photograph © courtesy of Hannes Freitag (FZE)

**Figure 3:**
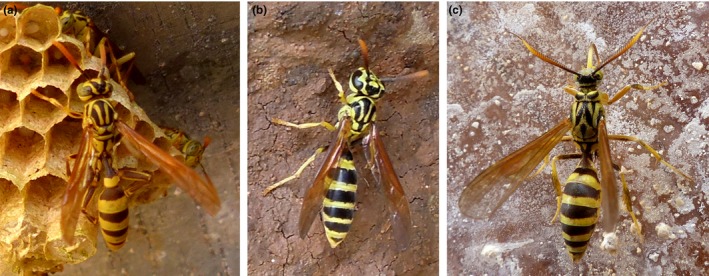
Two species of eusocial wasps and a “wasp‐moth” from Costa Rica—but which is which? The moth simulates not only the striped abdomen but also transparent and folded wings, petiolate abdomen, and patterned thorax of the wasps. Its true identity is revealed by its proboscis and pectinate antennae. (a) *Mischocyttarus* sp., (b) *Polybia* sp. (Hymenoptera: Vespidae), (c) *Pseudosphex laticincta* (Lepidoptera: Erebidae: Arctiinae)

**Figure 4:**
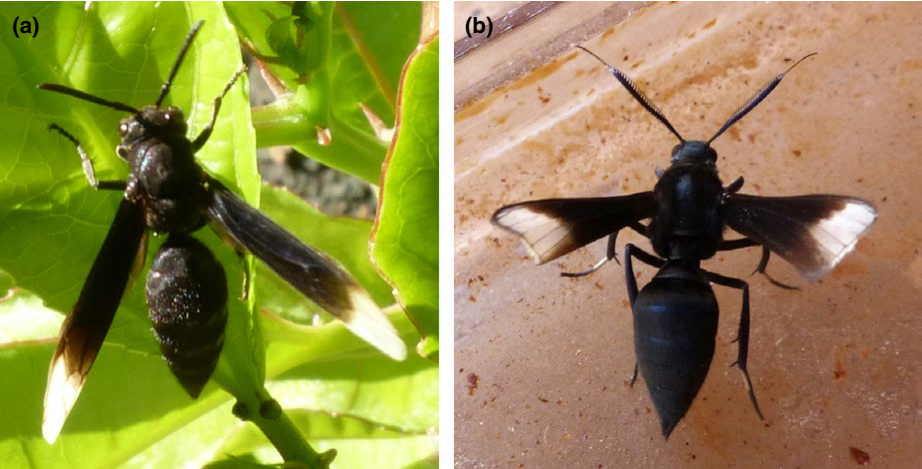
A case of accurate resemblance between a black eusocial wasp (a, Hymenoptera: Vespidae: *Parachartergus apicalis*) and a neotropical moth (b, Lepidoptera: Erebidae: Arctiinae: *Myrmecopsis strigosa*), showing the very same simulated features (abdomen, wings, petiole, thorax) discussed for yellow jackets (Figures 2 and 3). This exemplifies that the hypothesis discussed at length for yellow jackets can also be applied to understand accurate simulation of other color patterns. (The wing folding of the moth is incomplete in this photograph.)

The greatest diversity in fidelity probably occurs in the context of “wasp mimicry”. Stimulated by looking at wasp mimics in tropical communities and wondering in particular about the high degree of resemblance between particular Vespidae and certain arctiine moths (Lepidoptera; e.g., Figures 2, 3, 4), we started to doubt that Müllerian and Batesian mimicry *fully* explain the world‐wide syndrome of “wasp mimicry”. We asked specifically: *What selecting agent could drive highly accurate resemblance when inaccurate resemblance otherwise seems to suffice*?

Considering not the effects of adaptive resemblance (always an advantage for mimics) but rather the fidelity of the resemblance brings first the sensory abilities of the selecting agent(s) into focus, and then the evolutionary context in which they respond to an environmental stimulus as the main driving force in the evolution of adaptive resemblance. As Chittka and Osorio (2007) point out, the “cognitive dimensions of predator responses” are crucial—but we first need to establish which are the predators. In other words: *Who are the selecting agents, the drivers?*

## 3 Who are Selecting Agents for Wasp Resemblance?

So far, detailed studies on, for example, (co‐)occurrence of wasps and hoverflies have almost exclusively considered avian predators as selecting agents (Howarth & Edmunds, 2000; Howarth, Edmunds, & Gilbert, 2004; Kazemi, Gamberale‐Stille, Tullberg, & Leimar, 2014; Penney et al., 2012). However, not only vertebrates are insectivorous, but wasps also forage proteinaceous food for taking to the nest to feed the larvae, and necessarily kill large numbers of other insects.

We look at “wasp mimicry” independent of learning vertebrates as selecting agents and take account of three main characteristics of the wasps in question:

1. Yellow jackets are insect predators (and scavengers) that feed their larvae with insect meat.
2. Yellow jackets are eusocial insects, usually occurring in great abundance and at far higher densities than learning insectivorous predators, such as birds. (Colonies of polistine wasps can attain a million individuals: Zucchi et al., 1995.)
3. Yellow jackets should—and apparently do—visually recognize their nestmates (sisters) remote from the nest and usually do not attack them during foraging.

## 4 An Additional Interpretation of Wasp Mimicry

We propose the hypothesis that non‐aggression by wasps towards sisters during hunting is innate and on sight. This would not only be of advantage to all colony members but also to look‐alikes of other yellow jacket colonies and/or species. The widely observed similarity between species of unprofitable Vespidae thus might not (only) be driven by learning and educated vertebrates (Müllerian mimicry) but rather by the wasps themselves. Consequently, not only look‐alike predatory yellow jackets but also non‐hymenopterans such as moths, flies and other profitable prey would also benefit from being seen and treated as wasps. Thus, many wasp mimics might have evolved in order to avoid being eaten by predatory wasps rather than by educated vertebrate predators—and potentially without their involvement. As in Müllerian mimicry, look‐alike mimicking wasps do not deceive the model wasps (a mutualistic relationship) and—as in Batesian mimicry—profitable mimics deceive the selecting agents (Table 2).

Wasp resemblance in the traditional interpretation seems to represent a typical case of mimicry: a tripartite system of interactors comprising a model, a mimic and a selecting agent (Figure 1a). However, as outlined long ago (Wickler, 1968), the three component parts of a mimicry system do not necessarily involve or require three separate species (consider, e.g., “automimicry”, Brower, 1968), nor a shared type of predator. We also imagine the workers of a yellow jacket colony as a tripartite mimicry system: each and every individual is a defended or unprofitable potential prey item (model), while at the same time it is also a co‐mimic and a predator (selecting agent) (Figure 1b). Individuals from other conspecific wasp nests, or belonging to other predatory wasp species, have a mutual advantage if they share the same yellow jacket appearance; it is of advantage for all participating individuals. In this way, the close resemblance of different species of predatory wasps receives a simple interpretation without invoking learning predators as selecting agents, that is, Müllerian mimicry (learning being the key element of Müller's original theory). Thus, during hunting, the yellow jacket visual pattern acts as a stable “honest signal” for all yellow jackets. As discussed by Summers, Speed, Blount, and Stuckert (2015) in a different context, this is in effect because the interests of the would‐be predators and would‐be prey ‘align exactly’. Note that this wasp *masquerade* (see below; Table 1), with innately responding wasps as drivers, relates simply to inhibition of aggression with look‐alikes of oneself whereas wasp *mimicry* relates to the prey choice decisions of learning predators as drivers.

**Table 1:**
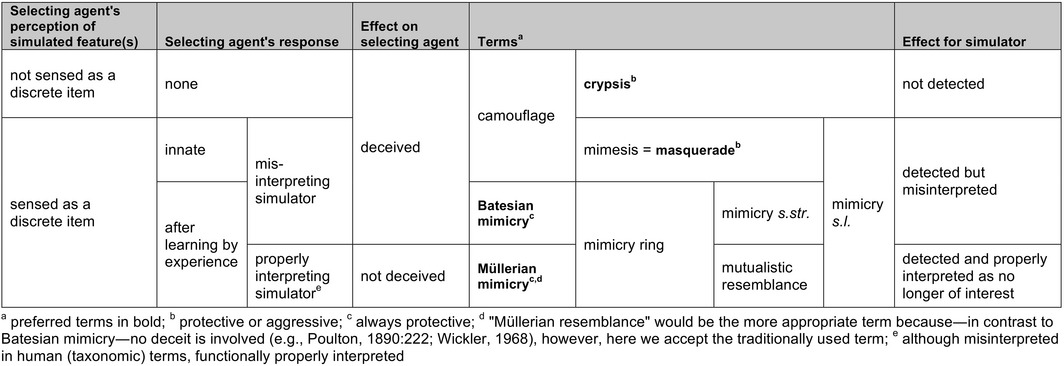
Categorizing and applying names to different types of adaptive resemblance—a problem in the past and in future. Looking at observations of adaptive resemblance from an evolutionary point of view, that is, focussing on the perspective of selecting agents, it is possible to define clearly four types (terms in bold); however, categorizing a specific example is often difficult or impossible because either the selecting agent(s) is not known or we do not know its sensory physiology. For example, there is a caterpillar (for humans) looking like a twig of a tree: If it is not sensed by the selecting agent as a discrete entity it is crypsis, if it is but innately misinterpreted as something uninteresting it is masquerade, and if it would only after experience with a stick be misinterpreted as uninteresting it would be Batesian mimicry. The effect is always the same (protection) but from an evolutionary perspective different causal mechanisms are involved. If we do not know the selecting agent(s) responsible (very often the case), we cannot understand and thereby meaningfully categorize our observation. If there are several selecting agents, they might have different sensory and neural abilities, and thus even several categories might apply. (A mantid simulating its environment (e.g., a flower) has dual advantages: It is not detected or is misinterpreted by predators as well as by potential prey—and crypsis and/or masquerade might both be implicated.) These difficulties should not permit us to forget about selecting agents but rather stimulate us to find out more about them. Unfortunately, in many cases the problem of unwarranted use of terms in publications will remain; in particular, the commonly used word camouflage is practically useless when studying evolution. Note that the typology presented here is independent of how a selecting agent responds, that is, being attracted or repelled, or (as in cases of deceiving a selecting agent) what the functional context of feature simulation is, that is, protection, predation, parasitism—these would make subtypes, as would the different sensory modalities (visual, chemical, mechanical) involved. For reviews and definitions in the context of adaptive resemblance see, for example, Wallace (1867), Carpenter and Ford (1933), Wickler (1968, 2013), Rettenmeyer (1970), Vane‐Wright (1976), Endler (1981), Pasteur (1982), Allen and Cooper (1985), Malcolm (1990), Starrett (1993), Komárek (2003), Ruxton, Sherratt, and Speed (2004), Stevens and Merilaita (2009, 2011), Skelhorn, Rowland, Speed, and Ruxton (2010), Skelhorn, Rowland, and Ruxton (2010), von Beeren, Pohl, and Witte (2012)

## 5 Simulating One's Own Potential Predator

Accepting our line of argument implies that “wasp mimicry” includes the possibility of simulating one's own potential predator: an essential intraspecific communication mechanism is the basis for evolution of *mutual* communication with other species having similar lifestyles (yellow jackets), while, at the same time, evolution of deception by profitable prey (e.g., mimicking moths) with different lifestyle becomes possible.

To date, a few cases of “sheep in wolves’ clothing”, involving insects or spiders, have been reported (Bates, 1862:509; Floren & Otto, 2001; Green, Orsak, & Whitman, 1987; Mather & Roitberg, 1987; Poulton, 1890:256; Rajashekhar & Siju, 2003; Rota & Wagner, 2006; Whitman, Orsak, & Green, 1988; Zolnerowich, 1992). Zaret (1977) studied a cannibalism‐inhibiting pattern element (“ocellus”) in a predatory cichlid fish and suggested that certain prey species might avoid being eaten as a result of their simulation of the ocellus; if so, he proposed to call this “predator mimicry”. As the evolutionary circumstances affecting this postulated system could hardly be more different to wasp simulation, we forbear to use his term (also used by Rota & Wagner, 2006), in particular as the terminology applied to mimicry *s.l*. remains far from clear‐cut, if not confusing (but see Table 1). Only considering Vespidae, there is no term available for our interpretation; considering Vespidae as selecting agents for profitable mimics, the term (predator) masquerade would be correct; by adding learning vertebrates Batesian and Müllerian mimicry are applicable, too (Table 2).

**Table 2:**
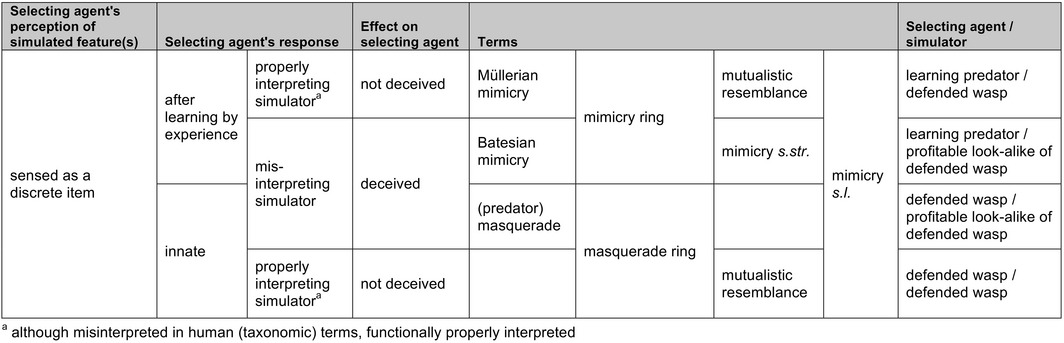
The additional interpretation of “wasp mimicry” as discussed in the text requires more than one category because two types of selecting agents are involved

## 6 Mimetic Fidelity—Wasps Plus Learning Vertebrates as Drivers

With respect to fidelity of mimicked features, several ideas suggest that eusocial wasps as selecting agents responding to simulations of their own patterns will be more discriminating than a guild of learning predators to their potential prey. First, consider that predators will be more or less discriminatory depending on their state of hunger, and the availability or not of alternative prey. Thus, a well‐fed insectivorous bird with abundant insects to choose among can readily afford to avoid inaccurate mimics, for fear they actually might be models (see Chittka & Osorio, 2007). For social wasps, in all circumstances, attacking a nest mate or another social wasp would be distractive and, perhaps, potentially even fatal. At the same time, there is an advantage in being able to discriminate themselves from profitable prey that mimic them—if ignored, they represent a loss of food resource. The two factors acting together would suggest that wasps should become very good at visual separation of ‘self’ (social wasps) from ‘similar nonselves’ (simulators).

The compromise hypothesis for inaccurate mimicry (Pekár et al., 2011) is also relevant here, insofar as a guild of vertebrate predators will collectively represent a wide range of sensory modalities and abilities, for which a ‘compromised’ or generalized mimetic similarity (“inaccurate mimicry”) may well be the best or most efficient solution. There is evidence of lack of precision in decision‐making by foraging birds (Kassarov, 2003). A key factor is the nature of perception itself. Do different predators, including birds, mammals, reptiles, amphibians and wasps, perceive yellow jacket patterns by some overall impression, as in Gestalt perception (Wagemans et al., 2012), or do they evaluate (and, perhaps summate) certain specific features (such as color, pattern, shape, sound, smell)? Feature‐integration theory (Treisman & Gelade, 1980) could offer an instructive alternative approach to Gestalt and appears relevant to the categorization hypotheses (Chittka & Osorio, 2007; Easley & Hassall, 2014). There is some evidence that dragonflies avoid wasps and wasp‐like flies based on yellow‐black stripes and, perhaps, shape (Kauppinnen & Mappes, 2003), but size also seems significant (Rashed, Beatty, Forbes, & Sherratt, 2005), for dragonflies at least.

While detailed research on social recognition in wasps (Cervo, Cini, & Turillazzi, 2015) demonstrates remarkable visual discriminatory abilities, cues used by wasps in the context of hunting and their specific roles have received insufficient, if any, attention. Unfortunately, we cannot learn from existing studies on visual and/or chemical self‐ and non‐self‐recognition as they were exclusively conducted in the context of the nest, at food sources, or in sexual interactions—thus in non‐comparable contexts. Vespidae forage solitarily; however, it is known that, at least in some species, the presence of conspecifics visually signals a food source (“local enhancement”, “social facilitation”: Parrish & Fowler, 1983; Fowler, 1992; Reid, MacDonald, & Ross, 1995; Slaa & Hughes, 2009; Pereira, Pirk, & Corley, 2016). Recognizing look‐alikes relates to either avoidance or attraction, context‐dependent during hunting and in the vicinity of a food source, respectively, thus―relevant to our hypothesis―look‐alikes are dismissed as potential prey.

If some vertebrates discriminate between profitable and unprofitable insects based on one or very few particular features (e.g., color, pattern, size or shape alone), and if particular members of a guild of insectivores dominate insect predation in particular habitats, this could lead to a variety of habitat‐specific mimics. From our ‘intellectualized’ evaluation of fidelity through examination of de‐contextualized corpses (which is what we do in museums, or when looking at photographs), such mimics might appear ‘inaccurate’—even though, in their natural habitat or in relation to relevant predators, they are able to achieve valuable protection. Perhaps all we can say at present is that similar‐looking and similarly sized social wasps, even if belonging to different genera of the Vespidae, are more likely to make use of very similar sensory abilities and neural processing compared to a guild of vertebrates in which the various members belong to different families and orders, differing in, for example, visual performance (Théry & Gomez, 2010), may rely on or prefer other sensory modalities, and vary in body size by one, two or even three orders of magnitude.

Although we have no reason to doubt the existence of typical mimicry rings involving eusocial wasps, at least in theory, wasp resemblance can be explained without the need for shared potential predators that mix up and reject both models and mimics after having had bad experience(s) with a model (as in Batesian and Müllerian mimicry). In other words, evolution of wasp resemblance could well be caused by two sorts of drivers: (1) foraging eusocial wasps that use an innate mechanism to recognize look‐alikes and inhibit preying on them, completely independent of vertebrate (learning) predators (Figure 1b vs. 1a). However, it seems likely that (2) general, visually oriented predators such as birds are *additional* selecting agents shaping similarity of other insects to wasps. Thus, in “wasp mimicry” *two sorts* of selecting agents (with different life‐styles) are plausibly acting. Then, the relative abundance of predatory wasps (individuals and species) that recognize look‐alikes as non‐food versus various predators that learn through experience could explain the accuracy and non‐accuracy of potentially profitable mimics. We would observe combinations of innate protective masquerade and learned Batesian and Müllerian mimicry, and recognize different sorts of selecting agents, namely those which respond innately and those which learn by experience. Thus, accurate mimics would be protected against both wasps and birds, whereas inaccurate mimics would be protected mainly against educated birds (which to a certain extent generalize a learned pattern) but not so well against wasps. In theory, proof could only come from studies in habitats where wasps prey on insects but learning predators do not occur—however, such places cannot be found. Anyway, our hypothesis focusses on an explanation of very accurate rather than the wide range of less accurate mimics; the basic message of our hypothesis is that wasps need to be considered not only as models but also as potential predators (thus as selecting agents) and that organisms accurately resembling Vespidae likely evolved as “sheep in wolves’ clothing” to avoid attack by their models.

From the many invertebrate predators known, for good reason, we have concentrated on wasps. However, other insect predators like asilid flies and dragonflies (Rashed et al., 2005) might also act as selecting agents; they need to be studied further.

## 7 Empirical Observations

The empirical basis for our suggestion that a wasp during hunting innately does not attack ‘that which looks like myself’ is the study of communities of models and mimics in tropical habitats. Superficially, several species and genera of day‐active arctiine moths (“wasp‐moths”; Lepidoptera: Erebidae: Arctiinae) very accurately resembling wasps that co‐occur with several co‐mimetic Vespidae of different genera (Figures 2, 3, 4) were observed at “El Bosque Nuevo”, Costa Rica, and “Panguana”, Peru. Several of these day‐flying moths simulate not only the yellow‐black color pattern but also the longitudinally folded forewings and, most strikingly, the petiole of the wasps (Kaye, 1913; Schrottky, 1909), involving extensive morphological re‐organization (Weller, Simmons, Boada, & Conner, 2000). In parallel, there are flies (Diptera) exhibiting similarly accurate resemblance to the wasps. We found yellow jacket wasps (naturally and experimentally) predating various moths but never yellow jackets or ‘accurate wasp‐moths’. Also, wasp abundance appeared much higher than that of insectivorous vertebrates. (Unfortunately, due to their low abundance in this habitat, for those species that inaccurately simulate wasps, no observations or experiments were possible so far.)

In support of our hypothesis also are observations of accurate simulation of several vespids with black abdomens and non‐transparent wings by arctiine moths (Figure 4)―they strictly parallel yellow jacket masquerade.

To date, structured studies on wasp resemblance do not deal with tropical mimetic assemblages but mainly address temperate hoverflies (Diptera: Syrphidae), many of which are considered to be Batesian mimics (e.g., Rotheray & Gilbert, 2011; cf. Figure 1)—although some work has questioned this assumption (Rashed & Sherratt, 2007). All studies have exclusively considered birds as selecting agents; strikingly, not only regarding hoverflies, yellow jackets have never been considered as potential predators even though they are widely recognized as potent general insectivores. The idea that the resemblance of hoverflies to yellow jackets offers potential protection against yellow jacket predation is expected to shed new light on the evolution of wasp mimicry, including the issue of number limitation that applies, in theory at least, to Batesian mimics, but arguably not to systems driven by innate responses. It should be mentioned that his data led Dlusski (1984) to the conclusion that sphecoid mimicry (mimicry between other insects and Hymenoptera; according to the terminology of Heikertinger, 1921) should be considered as a special form of mimicry, “significantly different from classic Batesian mimicry”.

## 8 Additional Complexity

As complex as the protective resemblance between wasps and other insects appears, it is yet more complex: several, if not all, arctiines which accurately simulate Vespidae (e.g., *Pseudosphex laticincta, Sphecosoma angustata*,* Myrmecopsis strigosa*) can be *un*profitable prey. As adults, they pharmacophagously take up pyrrolizidine alkaloids (PAs) from plants and sequester them as defensive metabolites (Boppré, 1995, and unpubl.), in some cases male‐biased, making them more or less unpalatable (details will be published elsewhere; for a general overview on PAs and pharmacophagy see Boppré, 2011). Also, some wasp‐moths as larvae seem to sequester defensive chemicals from hostplants (Boppré, unpubl.). While this news is very relevant for the classical interpretation of arctiine resemblance to wasps as it affects important questions, for example, on the (in)equality of defense in mimicry (Müllerian, Batesian or quasi‐Batesian mimicry) (Simmons & Weller, 2002), it does not change in principle the hypothesis discussed above.

## 9 Many Questions Remain

While the above‐mentioned additional complexity requires further study, our current ignorance does not invalidate the new interpretation given. Rather, this new perspective not only appears as plausible as the conventional interpretation but also generates subsidiary hypotheses for experimental testing, and makes apparent all kinds of relevant gaps in our knowledge. For example, it is often said “yellow jackets prey on insects”—but on which ones? And how do they detect, and how do they select prey? Generally, the subject of wasp mimicry and masquerade requires not only laboratory tests and modeling but in particular community approaches, extensive fieldwork and complex taxonomy; thus, it is a time‐consuming challenge. The subject demonstrates nicely today's continuing relevance of natural history studies (see Ricklefs, 2012). As clearly pointed out by Bates (1862:507), Schrottky (1909) as well as Kaye (1913) already more than a century ago, the striking resemblance between wasps and their mimics diminishes much after death (e.g., colors fade) and is often most obvious when the organisms concerned are encountered live, not as dead specimens set in a museum box. This and the need for data on syntopical occurrence makes studies based on collections of limited value, underlining the need for field work. If studies can be devised to investigate, in addition to structural and color features, behavioral characteristics (e.g., flight and activity patterns), and also sounds (cf. Gaul, 1952), the subject will gain additional biological realism as well as fascination.

Eventually, perhaps, it might turn out that in cases of adaptive resemblance there is great fidelity in simulated features when the selecting agent(s) fail to sense them (crypsis) or when innate behavior is involved (in cases of masquerade), while there is greater plasticity and less fidelity when selecting agents need to learn (in Batesian and Müllerian mimicry).

## Conflict of Interest

None declared.
